# Sugar-Sweetened Beverages and *Allergy Traits* at Second Year of Life: BRISA Cohort Study

**DOI:** 10.3390/nu15143218

**Published:** 2023-07-20

**Authors:** Alessandra Karla Oliveira Amorim Muniz, Elcio Oliveira Vianna, Luana Lopes Padilha, Joelma Ximenes Prado Teixeira Nascimento, Rosangela Fernandes Lucena Batista, Marco Antonio Barbieri, Heloisa Bettiol, Cecilia Claudia Costa Ribeiro

**Affiliations:** 1 Postgraduate Program in Public Health, Department of Public Health, Federal University of Maranhão—UFMA, Sao Luis 65020-060, Maranhao, Brazil; 2 Ribeirão Preto Medical School, University of São Paulo—USP, Ribeirao Preto 14049-900, Sao Paulo, Brazilmabarbieri@fmrp.usp.br (M.A.B.);

**Keywords:** atopic dermatitis, food allergy, allergic rhinitis, sugar-sweetened beverages, structural equation modeling, second year of life

## Abstract

Sugar-Sweetened Beverage (SSBs) consumption has risen in early life and it is plausible that it might increase children’s risk of allergies. In this paper, we analyzed the association of SSB consumption with allergies in children’s second year of life. This study analyzed data from a São Luís BRISA prenatal cohort in the follow-up of children (*n* = 1144) in their second year of life. *Allergy Traits* were a latent variable deduced from medical diagnoses of allergic rhinitis, atopic dermatitis, and food allergies. SSBs were investigated as a percentage of daily calories based on 24 h recalls, including industrialized fruit juices, soft drinks, and ready-made chocolate milk. Other variables analyzed were socioeconomic status, age, body mass index z-score, episodes of diarrhea, and breastfeeding. Our finds were that higher consumption of daily calories from SSBs was associated with higher *Allergy Trait* values (SC = 0.174; *p* = 0.025); older age (SC = −0.181; *p* = 0.030) was associated with lower *Allergy Trait* values; and episodes of diarrhea were correlated with *Allergy Traits* (SC = 0.287; *p* = 0.015). SSB exposure was associated with *Allergy Traits* in children’s second year of life; thus, abstaining from these beverages may also confer additional advantages in curtailing allergic diseases during early childhood.

## 1. Introduction

Sugar-sweetened beverages (SSBs) are the primary source of free sugars in individuals’ diets, significantly contributing to the energy density of the Western diet [1]. These beverages are those processed with added sugars, especially sucrose or high fructose corn syrup, and are represented by soft drinks, fruit flavored drinks, sweetened teas and coffees, energy drinks, and sweetened milks [2].

SSBs have been associated with epidemics of non-communicable diseases such as obesity, diabetes, and cardiovascular diseases [3]. Renowned international institutions have suggested limiting sugar intake to 5% of daily calories [4], 25 g/day for children and adolescents, and avoiding consumption until two years of age [5].

We have shown in BRISA Cohort analyses that consuming soft drinks during pregnancy is associated with childhood *asthma traits* in children’s second year of life [6]. Pregnant women’s soft drink consumption also increases early exposure to sugary items in their offspring [7]. Finally, higher sugar consumption by children is associated with *Asthma Traits* at two years of age, a latent variable composed of the following indicators: number of wheezing episodes, emergency care for wheezing, asthma diagnosis, and rhinitis diagnosis [8].

Higher sugar consumption during pregnancy can modulate children’s immunological responses, increasing their risk of allergic diseases. Allergic diseases comprise chronic immune-mediated diseases, which are more prevalent among children and are mainly represented by atopic dermatitis, allergic rhinitis, bronchial asthma, and food allergies [9].

Allergic diseases are a global public health problem, as they are highly prevalent and inappropriate care leads to complications and future NCDs, increasing morbidity, mortality, and health costs [10].

A systematic review with four cohort studies showed that sugar consumption during pregnancy was associated with greater chances of children developing allergic outcomes such as allergic rhinitis, atopy and eczema, wheezing, and food allergies up to seven years of age [11].

Sugar consumption causes advanced glycation end products (AGE), which can signal allergic pathways, triggering food allergies [12]. An animal model study showed the association between an AGE-causing diet and food allergy incidences [13]. AGE can also alter gut microbiota [14], and early-age gut dysbiosis seems to relate to the development of allergic diseases such as atopic dermatitis and food allergies [15].

Thus, it would be plausible that SSB exposure in a child’s early life may increase their risk of allergies. This subject is interesting since the consumption of these beverages has increased in early life. This study analyzed the association of SSB consumption with allergies in children’s second year of life. To represent allergies in young children, we tested the latent variable *Allergy Traits*, with evidence of validation in our previous study in the BRISA cohort [16], consisting of shared variance among atopic dermatitis, allergic rhinitis, and food allergy medical diagnoses.

## 2. Materials and Methods

Data from the Brazilian Ribeirão Preto and São Luís Birth cohort (BRISA) were analyzed for São Luís city in this study. São Luís is the capital of the state of Maranhão, located in one of the poorest regions of Brazil (Northeast), with a 0.768 Municipal Human Development Index [17]. The prenatal BRISA cohort followed its participants in three moments: baseline in the gestation period, first follow-up at birth, and second follow-up around the second year of life [18].

### 2.1. Study Population and Sample

At baseline, pregnant women who received prenatal care from the 22nd and 25th gestational weeks and agreed to participate were included (*n* = 1447). On the occasion of birth (1st follow-up), from May 2010 to November 2011, puerperal women (*n* = 1381) were interviewed again during the first 24 h after delivery. In the 2nd follow-up, mothers and children (*n* = 1151) were evaluated between September 2011 and March 2013. The study sample comprised 1144 mother–child pairs, considering follow-up losses [8,18] and the exclusion of non-respondents to the allergy outcome (Supplementary Figure S1).

### 2.2. Data Collection

The following information was used from the baseline questionnaire: household income (multiples of the Brazilian minimum wage; about BRL 510.00 in 2010), maternal schooling (in years), occupation of the head of the family, and economic class according to the Brazil Economic Classification Criteria—CEB [19] (D/E—poorer, C, and A/B—richer).

The following information from the second follow-up was used: children’s age (months), allergy diagnoses (atopic dermatitis, allergic rhinitis, and food allergies), total duration of breastfeeding, children’s length (cm) and weight (kg), and episodes of diarrhea in the previous two weeks.

The body mass index (BMI) z-score was evaluated based on the growth curves of the World Health Organization (WHO) [20], according to the age and sex of the children. The BMI z-score was used as a categorical variable, based on the WHO cut-off points for nutritional status: thinness, eutrophy, risk of overweight, overweight, and obesity [20].

Duration of breastfeeding was obtained from the following questions in the second follow-up: “Did the baby receive breast milk yesterday?” and “If not, until what age was the baby breastfed?” The variable was treated as categorical (did not breastfeed; ≤6 months; 6 to 12 months; 12 to 18 months; and >18 months of breastfeeding).

The investigation of *Allergy Traits* was based on the following questions in the follow-up questionnaire: “Since birth, has a physician diagnosed atopic dermatitis (eczema; intermittent skin allergy characterized by an intensely itching skin rash in any area of the body except around the baby’s eyes and nose or near their diaper)?”, “Has any doctor ever told you that your baby has allergic rhinitis?”, and “Has any physician ever told you that your baby has an allergy to any food?”.

Venous blood specimens collected were transferred to tubes containing anticoagulant ethylenediaminetetraacetic acid (EDTA) and utilized for determining eosinophil counts in children, expressed in cells per microliter (cells/μL).

#### Food Intake and Consumption of SSBs

The dietary intake of 733 children in the present study was assessed through interviews with their mothers and guardians, using 24-h dietary recall (24HDR), a valid assessment tool for analyzing habitual nutrient intake of the day before, as demonstrated in a previous study using the same population sample [21]. On the date of application of the recall, the youngest age presented by the children was 12 months (0.5%), and the oldest was 32 months (0.1%), with a mean age of 16.1 + 2.3 months.

The mother or guardian responded in detail about the foods and drinks consumed by the child, including the brand, method of preparation, portion size, or volume consumed, with the aid of a photo album.

The interviewers were trained in using the 24HDR, receiving a manual explanation of how to complete it. Training personnel performed a quality control step before inserting food consumption data into the program. Information on food and drink was collected and quantified in a standardized way. Subsequently, the Virtual Nutri Plus^®^ program (2010 version) converted consumption data into energy and nutrients. Then, the data were imported into Stata^®^ (version 15.0), where each child’s daily sugar intake was calculated. More details can be seen in a previous study [21].

Based on the reported foods and amounts, the total consumed energy per child and the percentage of calories from sugars added to beverages to this total daily intake was estimated. The consumption of SSBs was expressed as a percentage consumption of daily calories (%SSBs) and grams of added sugar ingested per day (grams/day), which were reported as a continuous variable.

Notably, the sugary drinks included sugar-sweetened beverages, not considering drinks sweetened with table sugar or alternative low- or no-energy sweeteners, such as aspartame and sucralose. Thus, the following SSBs were included in our analysis: industrialized fruit juices (excluding fresh fruit juice), soft drinks, and ready-made chocolate milk [8].

### 2.3. Statistical Analysis

Descriptive analyses were performed using Stata, version 15.0, in which continuous variables were represented as central tendency measures (mean and median) and categorical variables as relative and absolute frequencies.

Considering that the consumption data do not follow a normal distribution and that we had missing data, Plus software imputed values for the missing data based on the variables that preceded them in the path analysis, using frequency analysis and Bayesian analysis [22]. The Least Weighted Squares Mean and Variance adjusted -WLSMV was used as the model estimation method, because it is robust to non-normal and allows the imputation of missing data [23].

Two latent variables were included in our analysis: (a) socioeconomic status, composed of maternal schooling, occupation of the head of the family, household income, and economic class, to which we added [24] (b) *Allergy Traits*: a latent variable consisting of atopic dermatitis, allergic rhinitis, and food allergy medical diagnoses [16].

*Allergy Traits* were evaluated by confirmatory factor analysis (CFA). We analyzed the theoretical model through structural equation modeling (SEM) in the statistical software Mplus^®^, version 8 (Los Angeles, CA, USA). The following adjustment indices evaluated model fit: (a) *p* > 0.05 and confidence intervals above 90.0%, a root mean square error of approximation < 0.08 (RMSEA), and (b) the comparative adjustment (CFI) and Tucker–Lewis indices (TLI) (>0.95) [23]. We weighted the sample to follow-up losses, using it to estimate our structural equation models.

### 2.4. Structural Equation Modeling (SEM)

SEM is an epidemiological tool for testing a hypothetical causal structure of multiple observable and latent variables, minimizing measurement error in the estimation process. Latent variables are not observed but are derived from the combination of effect indicator variables, representing the common variance shared among them, resulting in an estimation of effects free from the bias originated by measurement errors. In the structural equation model, effect indicators are chosen to correspond to the theoretical definition of the concept: the latent variable [23].

Latent variables help capture complex phenomena that are challenging to measure directly, such as allergies in the first two years, reducing their measurement error. Advantageously, we have proposed *Allergy Traits*, with validation evidence in our previous study in the BRISA cohort [16].

**Figure 1:**
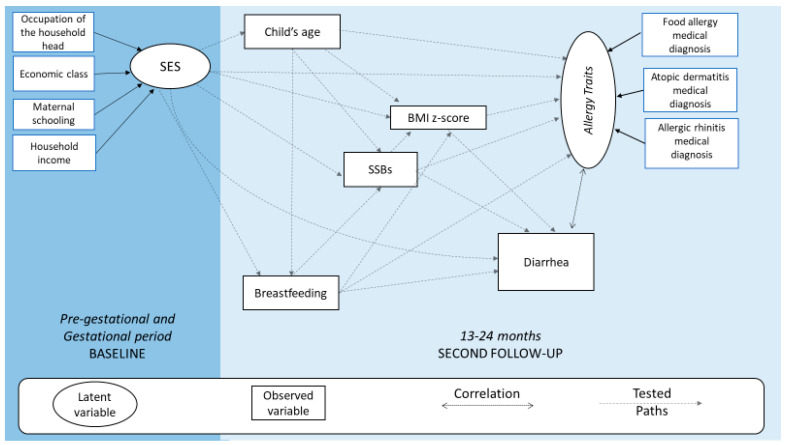
A conceptual model for analysis of the association between SSB consumption and *Allergy Traits* in children aged around two years. BRISA Prenatal Cohort, São Luís, Brazil, 2010–2013.

Figure 1 presents the conceptual model for the analysis of SSBs and *Allergy Traits* based on SEM.

Based on the literature, we constructed a hypothesis of our conceptual model: Socioeconomic Status (SES) will be the most distal variable, which is directly associated with the other variables of the model; SSBs will directly affect *Allergy Traits* around the second year of life [6,8,9,10,11,12] and these will also affected by the child’s age [25]; diarrhea will be correlated with *Allergy Traits* [26,27,28], and breastfeeding will be a protective factor for allergic diseases [29].

### 2.5. Consistency Analysis

In this study, we included an additional analysis to test the consistency of the association of SSBs with *Allergy Traits*. In this analysis, the eosinophil count replaced the diagnosis of allergic rhinitis (subjective measure). Previously, we had tested an allergic rhinitis compound in a latent *asthma trait* associated with SSBs in children in the second year of life [8].

Eosinophils were included as an outcome indicator because they actively participate in inflammatory responses, rising in allergic diseases [30]. Eosinophilia in the fourth week of life predicts the onset of atopic dermatitis in childhood, especially in individuals at high risk of atopy [31].

By introducing an objective measure predictive of allergic diseases replacing allergic rhinitis, we constructed an accurate indicator to identify if the association of SSBs would be a consistent result for *Allergy Traits.*

Furthermore, the association of other sugary products [8] and products referred to in the literature as allergens, such as milk and dairy products, were analyzed with *Allergy Traits* to assess our analysis’ consistency. Staple foods (fruits, vegetables, rice, leguminous plants, meats, fish, and shrimp) were also regressed in the models in their association with *Allergy Traits.*

### 2.6. Ethical Aspects

This study was approved by the Research Ethics Committee of the Federal University of Maranhão Hospital (protocol 4771/2008-30). All volunteers’ parents consented to participate in our research after being informed of its objectives.

## 3. Results

Table 1 shows the sociodemographic characteristics of the children’s families. Atopic dermatitis was very prevalent (10.3%) in the children’s second year of life. Eosinophilia was the most prevalent abnormality (one third had more than 580 cells/μL) (Table 2).

Our proposed model showed good fit for all analyzed parameters (Table 3). Atopic dermatitis (Factor Loading (FL) = 0.868; *p* = 0.002), food allergy (FL = 0.339; *p* = 0.012), and allergic rhinitis (FL = 0.255; *p* = 0.030) showed convergent factor loadings (Table 4).

Among the children, 14.5% already consumed SSBs, and 8.2% consumed more than the limit established by the WHO. On average, the children consumed 8.3% of their daily calories from SSBs (Table 5).

The higher percentage consumption of daily calories from SSBs was associated with higher values of *Allergy Traits* (Standardized Coefficient (SC) = 0.174; *p* = 0.025). Age increases reduced *Allergy Trait* values (SC = −0.181; *p* = 0.030). Episodes of diarrhea showed a correlation with *Allergy Traits* in children (SC = 0.287; *p* = 0.015) (Table 6).

Our consistency analysis showed no association between the percentage of daily calories from solid and pasty sugary products with *Allergy Traits.* Among the other products tested, only dairy consumption showed significant association, appearing as a protective factor for the *Allergy Traits* (Supplementary Table S2).

In the additional model of consistency analysis, which included eosinophil count as an indicator of latent outcome, the higher percentage consumption of daily calories from SSBs remained associated with higher values of *Allergy Traits* (SC = 0.223; *p* = 0.013).

## 4. Discussion

Early SSB exposure was associated with higher *Allergy Trait* values in children’s second year of life. Other sugary products failed to explain the allergy outcome.

*Allergy Traits* provided a good construct to assess for allergies in children’s second year of life due to three clinical indicators based on medical diagnoses (atopic dermatitis, allergic rhinitis, and food allergies) that had convergent factor loading. This approach proved advantageous as it can reduce measurement errors from any isolated allergic indicator, especially in children’s first years.

This is the first study to show early SSB exposure and allergy indicators in children’s second year of life. An analysis of children as young as six years and adolescents showed that consuming drinks with excessive free fructose ≥ 5 times/week increased their chances of allergic sensitization by 2.5 times as compared to consuming less than 3 times a month [26]. In a previous study, we showed the association between early SSB exposure and *Asthma Traits* in children’s second year of life [8]. The clinical indicators we proposed for the latent variables *Asthma Traits* (rhinitis and wheezing episodes) and *Allergy Traits* may be expected in individuals’ allergic march.

**Figure 2:**
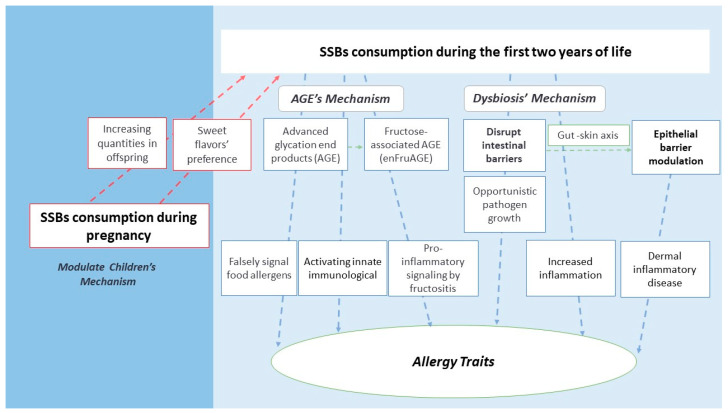
Plausible Mechanisms to association of early exposure to SSBs and allergies.

The explanation for the association between SSB exposure and allergies especially involves three main mechanisms: *Modulate Children’s Mechanism*, *AGE’s Mechanism*, and *Dysbiosis’ Mechanism* (Figure 2).

A higher consumption of SSBs during pregnancy resulted in a higher percentage of calories from sugary products in children at two years of age [7]. These findings involve genetic and environmental alterations, which can lead to a preference for sweets due to changes in taste, as well as the ingestion of greater amounts of sugars in offspring [27].

Increased consumption of SSBs by children can lead to increased oxidative stress, AGE, inflammatory response, gut dysbiosis, exacerbation of immune response, and increased *Allergy Traits*. The *AGE’s Mechanism* can involve (1) high concentrations of AGE-forming sugars that could falsely signal food allergens, activating innate immunological responses and developing early food allergies [12]; (2) high AGE levels that can trigger immunological responses by the Th2 pathway [13]; and (3) an excess of unabsorbed free fructose and the alkaline intestinal medium that could form fructose-associated AGE (enFruAGE) and pro-inflammatory signaling by fructositis, leading to large mucus production and respiratory problems [14,28].

Early exposure to sugar leads to structural and functional changes in the microbiome (dysbiosis) and in the production of inflammatory cytokines [25]. Another pathway of the dysbiotic mechanism is associated with increased intestinal permeability in the presence of fructose, favoring the colonization of pathogenic microorganisms, the dysregulation of the immune system, and the development of food allergies [15,32]. In general, opportunistic pathogen growth, altered metabolic profiles, and increased inflammation may explain how gut microbiome alterations affect hosts’ health [33].

The gut microbiota regulates immune responses, affecting sites far from the gastrointestinal tract, such as the gut–skin axis. Thus, the intestine–skin axis represents the interaction of the intestine in epithelial barrier modulation, in which the cytokines and cells it produces begin to act on the skin [34,35]. An in vitro study showed that changes in intestinal permeability could increase cytokines and dermal inflammatory disease signals, accumulating fatty acids in the skin and reducing its functionality [36].

In this study, diarrhea was correlated with *Allergy Traits*. Diarrhea can be a symptom of food allergies [37] and characterizes gut dysbiosis [38], which can precede allergic disease and increase the risk of its development [15,39].

Children with allergies (especially to food) show diarrhea episodes [40] and elimination diets up to their second year of life, reducing nutrient absorption and the weight gain [41]. The relationship between obesity and allergies has been shown in older children and adolescents [42]; moreover, the effect of SSBs on excess weight is not observed in the second year of life, but at more advanced ages [7,43]. The BMI z-score by age was not associated with *Allergy Traits* in our study, but it is possible that this effect is observed in older children.

Age increase (in months) reduced *Allergy Trait* values. Immune tolerance to antigens begins during pregnancy and continues throughout a child’s first years. During this period environmental factors, including nutrition, can permanently alter and irreversibly the damage the immune system [44]. The window of immunity has been defined as a window of opportunity and also of susceptibility and comprises the period of the first thousand days of life [45]. Thus, ingesting the first allergenic proteins in the diet configures exposures that help modulate immune responses and reduce the risk of allergies [46].

Unexpectedly, our consistency analysis showed dairy consumption as a protective factor for the *Allergy Traits.* Children diagnosed with food allergies and atopy may already have diets that exclude milk and its derivatives. Dairy products can also elevate butyrate levels, which can act on the immune system by regulatory T-cell expansion and are related to beneficial bacterial colonization [47]. Dietary fiber may also raise butyrate levels; however, our results showed no association with *Allergy Traits*.

As an additional analysis, the dairy consumption with *Allergy Traits* lost significance when we removed the food allergy indicator from the latter. These results support that apparent protection had been biased because children diagnosed with food allergies typically excluded milk and its derivatives.

By replacing allergic rhinitis with eosinophil count in the consistence analysis, we included a more objective measure of our studied outcome, provided greater consistency to association of SSBs with *Allergy Traits*, and avoided attributing the weight of the association to allergic rhinitis, which we tested in a previous study to *Asthma Traits* [8]. This study estimated the association between beverages rich in added sugars and the latent variable “child *asthma traits*” in the second year of life, and similarly to what was verified in the present study, the authors showed that a high percentage of daily calories from added sugars to SSBs was directly associated with higher values of childhood *asthma traits*, without mediation pathways [8].

We can list the lack of allergenic tests in the BRISA Cohort, and the allergy indicators based on a questionnaire, in which the parents reported diagnosis of allergic diseases as limitations of this study. Circumventing them, we analyzed this outcome as a latent variable based on the variance between three indicators, reducing measurement errors. As a strength, equation modeling enabled us to explore multiple exposures, including other dietary items associated with *Allergy Traits*.

Although the lack of other studies evaluating the association of SSBs with allergic diseases in the same age group configures innovation, it hindered the comparability of our data in different populations. An age group that should not be exposed [5] to sugar consumption showed a worrying incidence rate, and the percentage of children with early exposure to SSBs can be compared to data from American children.

In US children aged 0–5 years, SSBs were consumed by 5% of children aged 6 to 11 months and 31% of children aged 12 to 23 months, and the caloric contribution of these beverages concerning the intake total was 3.4% [48]. The introduction of SSBs has been frequent and begins early in childhood, even before six months. Introducing these drinks during pregnancy and in the first six years of life determines the preference for sweet flavors and their intake in increasing quantities later [27]. Thus, the consumption of SSBs ceases to be an option included by parents that belong to children’s choices and preferences.

Allergies and asthma feature among the immune-mediated non-communicable diseases associated with increased future risk of other such diseases [49,50]. The origins of immune-mediated diseases have been explained by the early exposure of immature immunological systems [51].

## 5. Conclusions

We showed that SSB exposure is associated with *Allergy Traits* in children’s second year of life, similar to what we already pictured for *Asthma Traits*. This latent variable does not aim at clinical implementations in allergy diagnosis; instead, it is possible for epidemiological studies, which, like ours, may identify factors associated with greater consistency to *Allergy Traits*, to encourage prevention strategies in the health care of newborns and children.

## 6. Clinical Implications

International guidelines have cautioned against consuming SSBs in children under two years to mitigate the risk of obesity and non-communicable diseases in later life. Our findings suggest that early non-exposure to SSBs may also reduce immune-mediated diseases. Abstaining from these beverages may also confer additional advantages in curtailing allergic diseases during early childhood. Our findings suggest that coping with these immune-mediated diseases should focus on the economic and commercial determinants that increase such early exposure to SSBs.

## Figures and Tables

**Table 1:** Family’s sociodemographic characteristics in the baseline. BRISA Prenatal Cohort. São Luís, Brazil, 2010–2013.

Variable	Baseline	
Maternal years of schooling	*n*	%
0–4	17	1.5
5–8	114	10.0
9–11	872	76.2
≥12	126	11.0
No information available	15	1.3
Occupation of the head of the family		
Unskilled manual workers	308	26.9
Semi-skilled manual workers	457	39.9
Skilled manual workers	51	4.5
Office positions	161	14.1
Higher education professionals	57	5.0
Administrators/owners	36	3.1
No information available	74	6.5
Household income ^1^		
<1	12	1.1
1 and <3	521	45.5
3 and <5	360	31.5
≥5	207	18.1
No information available	44	3.8
Economic class		
D-E	169	14.8
C	743	64.9
A-B ^2^	166	14.5
No information available	66	5.8
Total	1144	100

^1^ Number of minimum wage. ^2^ A and B are the highest family incomes.

**Table 2:** Demographic and health characteristics in children’s second year of life. BRISA Prenatal Cohort, São Luís, Brazil, 2010–2013.

Variable	2nd Year of Life	
Age (months)	Mean	SD ^1^
	16.0	2.3
	Median	Percentiles 25–75
	15.0	14.0–17.0
Gender	*n*	%
Male	571	49.9
Female	568	49.6
No information available	5	0.4
Duration of breastfeeding		
Did not breastfeed	8	0.7
≤6 months	157	13.7
6 to 12 months	126	11.0
12 to 18 months	720	63.0
>18 months	70	6.1
No information available	63	5.5
BMI z-score ^2^		
Thinness	21	1.8
Eutrophy	691	60.4
Risk of overweight	289	25.3
Overweight	98	8.6
Obesity	32	2.8
No information available	13	1.1
Physician made diagnosis of allergic rhinitis		
No	1071	93.6
Yes	73	6.4
Physician made diagnosis of atopic dermatitis		
No	1026	89.7
Yes	118	10.3
Physician made diagnosis of food allergies		
No	1112	97.2
Yes	32	2.8
Eosinophil count (tertile)		
1st tertile (13–311 cells/μL)	255	22.3
2nd tertile (312–580 cells/μL)	255	22.3
3rd tertile (>580 cells/μL)	255	22.3
No information available	379	33.1
Total	1144	100

^1^ SD: standard deviation. ^2^ z-score of Body mass index for age and sex.

**Table 3:** Indexes of model fit by structural equation model to analyze association between *Allergy Traits* and SSB consumption in children’s second year of life. BRISA Prenatal Cohort, São Luís, Brazil, 2010–2013.

Indicators	Model
Root Mean Square Error of Approximation (RMSEA)	0.017
RMSEA (90% CI ^1^)	0.000–0.028
*p*-value	1.0
Comparative Fit Index (CFI)	0.973
Tucker-Lewis Fit Index (TLI)	0.956

^1^ Confidence Interval.

**Table 4:** Standardized coefficients, standard errors, and *p*-values of latent variable *Socioeconomic Status* and *Allergy Traits*. BRISA Prenatal Cohort, São Luís, Brazil, 2010–2013.

Latent Variable	Indicator Variables	Factor Loading	SE ^1^	*p*-Value
*Socioeconomic status (SES)*	Occupation of the head of the family	0.533	0.031	<0.001
	Household income	0.623	0.034	
	Maternal schooling	0.432	0.030	
	Economic class	0.678	0.031	
*Allergy Traits*	Atopic dermatitis	0.868	0.279	0.002
	Allergic rhinitis	0.255	0.117	0.030
	Food allergy	0.339	0.135	0.012

^1^ SE = Standard Error.

**Table 5:** Consumption of SSBs and other products with added sugar data (daily) in children’s second year of life. BRISA Prenatal Cohort, São Luís, Brazil, 2010–2013.

Variable		Mean	SD ^1^	Median	Percentiles 25–75
Consumption of sugar-sweetened beverages	%	8.3	6.8	5.9	3.4–11.6
	g	20.0	13.6	16.5	10.5–23.0
Chocolate milk (Kcal)		120.3	91.8	88.2	44.1–180.7
Soft drinks (Kcal)		56.4	27.6	50.3	38.7–68.4
Industrialized juices (Kcal)		75.8	51.7	63.0	45.0–87.0
Consumption of sugared pasty products	%	11.5	6.8	10.0	6.6–13.8
	g	20.5	12.1	19.8	9.9–22.0
Dairy drinks (Kcal)		124.8	68.7	131.7	65.8–131.7
Popsicles and ice cream (Kcal)		172.7	62.2	193.7	110.5–221.0
Industrialized baby food (Kcal)		75.8	13.7	76.0	73.0–76.0
Consumption of sugared solids	%	7.5	5.6	5.8	3.9–9.7
	g	15.4	12.3	10.9	7.2–18.1
Cakes (Kcal)		121.6	94.9	95.3	47.7–214.5
Cookies (Kcal)		90.3	81.3	63.7	42.5–90.0
Percentage of sugar intake in products with high sugar in relation to total calorie					
SSBs		*n*		**%**	
	None	627		85.5	
	≤5%	46		6.3	
	>5%	60		8.2	
Sugared pasty products	None	404		55.1	
	≤5%	35		4.8	
	>5%	294		40.1	
Sugared solids	None	678		92.5	
	≤5%	23		3.1	
	>5%	32		4.4	
All products with high sugar	None	310		42.3	
	≤5%	63		8.6	
	>5%	360		49.1	
Total		733		100	

^1^ SD = Standard Deviation.

**Table 6:** Adjusted model indicators and standardized estimates of direct effects considering the association between variables and *Allergy Traits* in children’s second year of life. BRISA Prenatal Cohort, São Luís, Brazil, 2010–2013.

Exposures	Standardized Estimates of the Direct Effect on *Allergy Traits*		
	SC ^1^	SD ^2^	*p*-Value
Child’s Age (association)	−0.181	0.083	0.030
Higher percentage consumption of daily calories from SSBs (association)	0.174	0.078	0.025
Episodes of diarrhea (correlation)	0.287	0.118	0.015

^1^ SC = Standardized Coefficient; ^2^ SD: standard deviation.

## Data Availability

The data that support the findings of this study are available on request from the corresponding author. The data are not publicly available due to privacy or ethical restrictions.

## Supplementary Materials

The following supporting information can be downloaded at: https://www.mdpi.com/article/10.3390/nu15143218/s1, Figure S1: Flow diagram of the BRISA prenatal cohort study, São Luís, Brazil title; Table S1: Food consumption data (daily) in children’s second year of life. BRISA Prenatal Cohort, São Luís, Brazil, 2010–2013; Table S2: Adjusted model indicators and standardized estimates of direct effects considering the association between food consumption variables and *Allergy Traits* in children’s second year of life. BRISA Prenatal Cohort, São Luís, Brazil, 2010–2013.
