# Supplementary material for: Sugar-Sweetened Beverages and Allergy Traits at Second Year of Life: BRISA Cohort Study
Source: Nutrients. 2023 Jul 20;15(14):3218. doi: 10.3390/nu15143218 (PMC10383806; doi:10.3390/nu15143218)
Supplement: Supplementary file 1 [file nutrients-15-03218-s001.zip › nutrients-2457843-supplementary.pdf]

SUPPLEMENTAL FIGURE

Figure S1. Flow diagram of the BRISA prenatal cohort study, São Luís, Brazil.

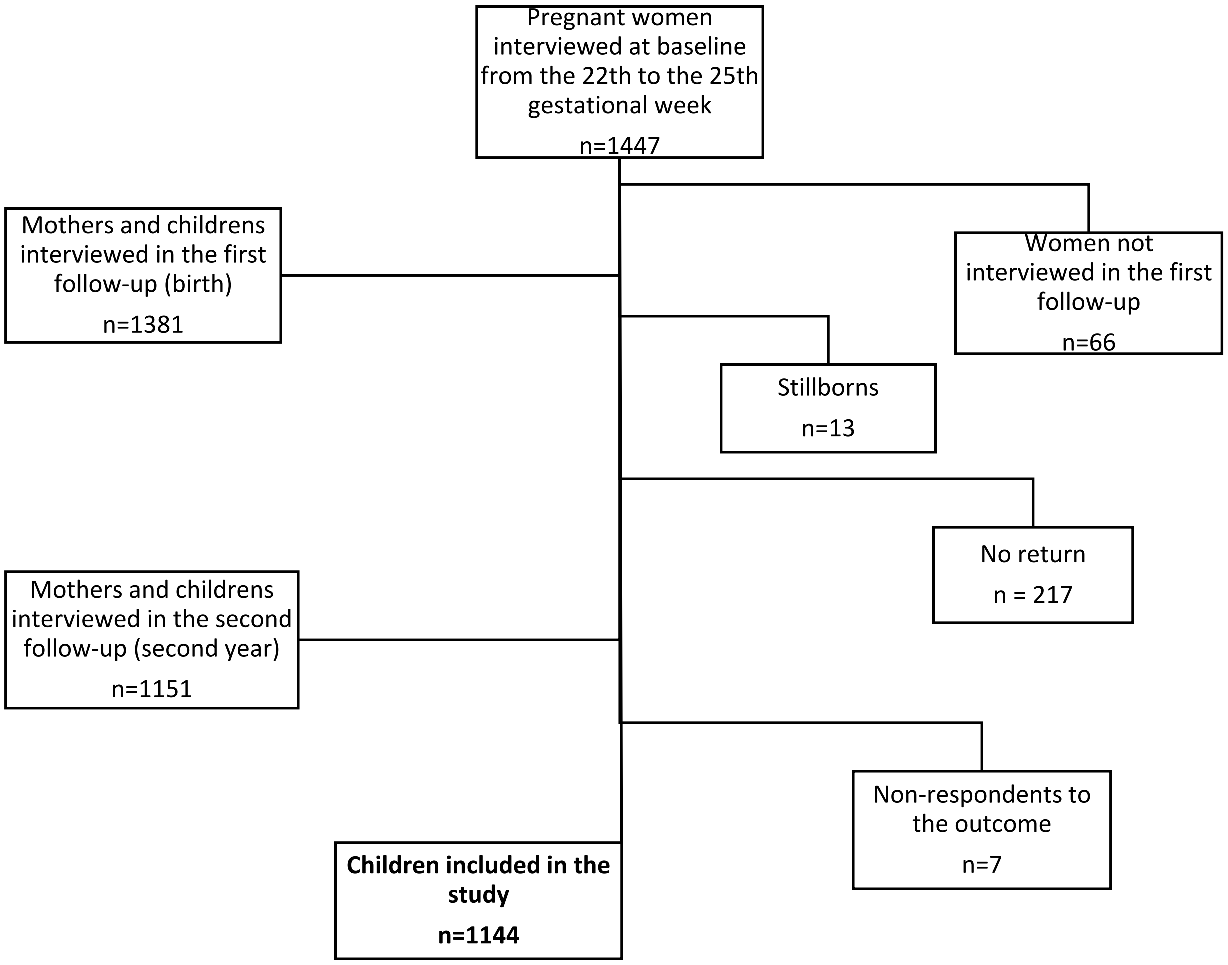

SUPPLEMENTAL TABLES

Table S1. Food consumption data (daily) in children's second year of life. BRISA Prenatal Cohort, São Luís, Brazil, 2010-2013**.**

Staple foods	Mean	SD[1]	Median	Percentiles 25-75
Fruits (Kcal)	83.8	66.1	68.5	41.1-103.0
Vegetables (Kcal)	123.7	109.5	97.1	54.9-164.4
Rice (Kcal)	115.8	69.8	112.2	62.3-149.6
Bean (Kcal)	50.9	83.6	31.1	20.7-53.2
Meat (Kcal)	149.3	107.5	116.4	81.4-196.8
Fish and shrimp (Kcal)	187.6	138.6	143.6	96.0-269.2
**Other products**				
Dairy products (Kcal)	360.5	252.0	317.4	158.7-476.2
Infant Formulas (Kcal)	332.8	301.5	243.9	144.5-411.1

[1] SD: standard deviation

Table S2. Adjusted model indicators and standardized estimates of direct effects considering the association between food consumption variables and Allergy Traits in children's second year of life. BRISA Prenatal Cohort, São Luís, Brazil, 2010-2013.

**Association of Allergy Traits with the energy percentage of**	**Standardized estimates of the direct effect**			**Adjusted model indicators**				
	**SC[1]**	**SD[2]**	**p-value**	**RMSEA[3]**	**RMSEA (90% CI[4])**	**p-value**	**CFI[5]**	**TLI[6]**
Pasty sugary products	0.024	0.061	0.692	0.017	0.000-0.029	1.000	0.962	0.936
Solid sugary products	-0.083	0.089	0.355	0.021	0.007-0.031	1.000	0.963	0.935
Fruit	0.132	0.078	0.089	0.022	0.010-0.032	1.000	0.956	0.926
Vegetables	0.049	0.066	0.451	0.019	0.003-0.030	1.000	0.969	0.948
Rice	0.050	0.098	0.608	0.023	0.012-0.034	1.000	0.960	0.931
Leguminous plants	0.003	0.065	0.959	0.015	0.000-0.027	1.000	0.978	0.963
Meat	0.046	0.074	0.533	0.023	0.011-0.033	1.000	0.955	0.924
Fish and shrimp	-0.006	0.062	0.928	0.021	0.008-0.032	1.000	0.959	0.930
**Dairy products**	-0.381	0.095	**<0.001**	0.015	0.000-0.027	1.000	0.981	0.969
Infant formulas	0.019	0.071	0.785	0.015	0.000-0.027	1.000	0.979	0.964

[1] SC=Standardized Coefficient; [2] SD: standard deviation; [3]RMSEA= Root Mean Square Error of Approximation; [4]CI= Confidence Interval; [5] CFI= Comparative Fit Index; [6]TLI=Tucker-Lewis Fit Index
